# UPF1 silenced cellular model systems for screening of read-through agents active on β^0^39 thalassemia point mutation

**DOI:** 10.1186/s12896-018-0435-0

**Published:** 2018-05-15

**Authors:** Francesca Salvatori, Mariangela Pappadà, Giulia Breveglieri, Elisabetta D’Aversa, Alessia Finotti, Ilaria Lampronti, Roberto Gambari, Monica Borgatti

**Affiliations:** 1 Department of Life Sciences and Biotechnology, Biochemistry and Molecular Biology Section, University of Ferrara, Via Fossato di Mortara 74, 44121 Ferrara, Italy; 2 Biotechnology Center, University of Ferrara, Ferrara, Italy

**Keywords:** Read-through activity, UPF1, Nonsense-mediated mRNA decay, β thalassemia, Single point mutation

## Abstract

**Background:**

Nonsense mutations promote premature translational termination, introducing stop codons within the coding region of mRNAs and causing inherited diseases, including thalassemia. For instance, in β^0^39 thalassemia the CAG (glutamine) codon is mutated to the UAG stop codon, leading to premature translation termination and to mRNA destabilization through the well described NMD (nonsense-mediated mRNA decay). In order to develop an approach facilitating translation and, therefore, protection from NMD, ribosomal read-through molecules, such as aminoglycoside antibiotics, have been tested on mRNAs carrying premature stop codons. These findings have introduced new hopes for the development of a pharmacological approach to the β^0^39 thalassemia therapy. While several strategies, designed to enhance translational read-through, have been reported to inhibit NMD efficiency concomitantly, experimental tools for systematic analysis of mammalian NMD inhibition by translational read-through are lacking.

**Results:**

We developed a human cellular model of the β^0^39 thalassemia mutation with UPF-1 suppressed and showing a partial NMD suppression.

**Conclusions:**

This novel cellular model could be used for the screening of molecules exhibiting preferential read-through activity allowing a great rescue of the mutated transcripts.

**Electronic supplementary material:**

The online version of this article (10.1186/s12896-018-0435-0) contains supplementary material, which is available to authorized users.

## Background

Nonsense mutations are among the most important alterations of the β globin gene in β thalassemia patients and are based on a change of an amino acid codon to a premature termination codon (PTC). For instance, in β^0^39 thalassemia the CAG (glutamine) codon of the β globin mRNA is mutated to the UAG stop codon [1, 2], leading to premature translation termination and to mRNA destabilization through the well described NMD (nonsense-mediated mRNA decay) [3, 4]. Several ribosome-binding drugs, including select aminoglycosides and synthetic novel small molecules, induce ‘translational read-through’ of PTCs, restoring full-length functional protein in a number of preclinical and clinical settings [5]. For example, different aminoglycoside antibiotics are known since many years to be able to suppress nonsense mutations, exerting their activity by binding at the decoding center of the eukaryotic ribosome, thereby altering the ability of translation termination factors to accurately recognize a PTC [6, 7]. We have reported that the aminogycoside G418 is able to induce read-though of the β^0^39 globin mRNA in erythroid precursor of homozygous β^0^39 thalassemia patients, allowing production of HbA [8]. However this effects was not very high, probably due to the fact that mRNAs bearing PTCs (such as the β^0^39 globin mRNA) display decreased stability due to the well known nonsense mediated decay (NMD) activity. This is a rather general feature of PCTs carrying mRNAs and is acknowledged as the major reason of the low activity of several read-through molecules. Of great interest is the recent identification and characterization of compounds that do not show read-through activity in human cells when used as single agents but that strongly potentiate the read-through activity of aminoglycoside antibiotics by a still unclear mechanism of action [9]. This study is the proof-of-principle that the two mechanisms (i.e. read-thorugh and read-through potentiation) are clearly separated. Among the possible read-though potentiators are molecules which inhibit NMD [10]. Therefore the development of a NMD deficient experimental model system might be of interest to increase the screening efficiency of novel read-though molecules. The objective of this study is to develop a K562 based cellular model system lacking efficient NMD, allowing improved screening of read-through molecules. The K562 cell line [11] is well known as a useful experimental model system to study the expression of embryo-fetal globin genes, as well as their modulation [12, 13]. Interestingly, especially for reaching the aims of the present study, K562 cells express the β globin gene at very low levels both in their uninduced state as well as after erythroid differentiation stimulated by a variety of chemical inducers, such as tallimustine [14], mithramycin [15] and rapamycin [16]. In the present paper, we report the development of K562 cell lines integrating multiple copies of the human β globin gene carrying the β^0^39 thalassemia mutation. To this aim we used K562 clones harbouring β^0^39 or β globin genes produced previously in our laboratory [17]. We induced in these cells knockdown of UPF1, one of the major factor of NMD, with the objective of generating a NMD-deficient cell line and treated them with G418, a well known read-through molecule.

## Methods

### Cell cultures

The human leukemia K562 [11] (ATCC, VA, USA) and K562-derived cells (obtained modifying K562 as reported in Salvatori et al., 2009 [8]) were cultured in humidified atmosphere of 5% CO_2_/air in RPMI 1640 medium (SIGMA, St Louis, MO, USA) supplemented with 10% fetal bovine serum (FBS; Biowest, Nuaillè France), 50 units/mL penicillin and 50 mg/mL streptomycin (Cambrex Biowhittaker Europe) (Lozzio & Lozzio, 1975 [11]). Cell growth was studied by determining the cell number per ml by ZF Coulter Counter (Coulter Electronics, Hialeah, FL, USA).

### Production and characterization of K562-derived cell clones by UPF1 shRNA lentivirus vectors

Two lentiviral constructs were used to transduce K562-derived cells (β^0^39.m5 and βwt.wt3 clones [8]) to induce the expression of shRNA against UPF1 or scramble. These vectors carry two LTR sequences, the SV40 origin of replication and the sequence of the shRNA, against UPF1 or scramble, under the control of the U6 promoter. shRNA and U6 promoter sequences were obtained from commercial retroviral vectors: pRS-shRNA-UPF1 and pRS-shRNA-scramble (Origene Technologies, Rockville, MD, USA). The lentiviral constructs were transfected into packaging cells with helper vectors, useful to produce lentiviral particles, which were subsequently titered and used to infect β^0^39.m5 and βwt.wt3 cells. These cells were then cloned by limiting dilutions and positive clones were identified by PCR. Treatment with G418 was carried out by adding the appropriate drug concentrations at the beginning of the experiment (cells were usually seeded at 30.000 cells/ml). The medium was not changed during the induction period.

In particular, K562-derived cell clones were collected by centrifugation at 1.200 rpm for 5 min at 4 °C, washed with PBS and lysed using 0.5% SDS and 0.5 mg/ml Proteinase K. The genomic DNA was than purified by phenol – chloroform extraction. To quantify the amount of lentiviral vectors in the genomic DNA we used 100-200 ng of template and a standard curve of normal control DNA in the range 50-300 ng. PCR primers and probe for the lentiviral vector were: LV forward primer, 5′-GGA TCT CGA CGG TAT CGG TTA ACT-3′, LV reverse primer, 5′-GGG TGT GTG CCC AGA TGT TCT-3′, LV probe, 5’AGC TCT CTC GAC GCA GGA CTC GGC-3′. The fluorescent reporter and the quencher were: 6-carboxyfluorescein (FAM) and 6-carboxy-N,N,N′,N′-tetramethylrhodamine (TAMRA), respectively. For real-time PCR analysis we used as reference gene the ^A^γ globin gene. PCR primers and probe were: ^A^γ globin forward primer, 5′-TGG CAA GAA GGT GCT GAC TTC-3′, ^A^γ globin reverse primer, 5’-TCA CTC AGC TGG GCA AAG G-3′, ^A^γ globin probe, 5’-FAM-TGG GAG ATG CCA TAA AGC ACC TGG-TAMRA-3′. The probe, was fluorescently-labeled with FAM (Applied Biosystems). Quantitative real-time PCR assay was carried out using two different instruments: iQ™5 Multicolor Real-Time PCR Detection System and CFX96 Touch™ Real-Time PCR Detection System (Bio-Rad Laboratoires, Hercules, CA, USA).

On the contrary, to extract RNA from the clones obtained by transduction with lentiviral vector, cells were collected by centrifugation at 1.200 rpm for 5 min at 4 °C, washed with PBS, lysed with 1 ml of TRIZOL^®^ Reagent (Thermo Fisher Scientific, Waltham, MA, USA), according to the manufacturer instructions. For gene expression analysis 1 μg of total RNA was reverse transcribed by using random hexamers. Quantitative real-time PCR assay were performed as described elsewhere [17]. For quantitative real-time PCR analysis of UPF1 mRNA, we used the human UPF1 expression assay kit (Thermo Fisher Scientific, Waltham, MA, USA); while for real-time PCR analysis of β globin mRNA, the following primers were used: β globin forward primer, 5’-CAA GAA AGT GCT CGG TGC CT-3′; β globin reverse primer, 5’-GCA AAG GTG CCC TTG AGG T-3′; βglobin probe, 5’-FAM-TAG TGA TGG CCT GGC TCA CCT GGA C-MGB-3′. The endogenous control human GAPDH, whose cDNA was amplified using human GAPDH expression assay kit (Thermo Fisher Scientific, Waltham, MA, USA), was used as reference gene. The fluorescent reporter and the quencher of UPF1, β globin and GAPDH probes were FAM and MGB, respectively.

### FACS analysis

K562-derived cells treated with G418 were permeabilized and marked with the antibody against β globin using the Cytofix/Citoperm™ Kit (BD Biosciences Pharmingen, Franklin Lakes, NJ, USA). 1.5 × 10^6^ cells were first washed with 500 μl of PBS 1X (Phosphate-Buffered Saline*,* Cambrex, NJ, USA) and then incubated with 500 μl of BD Cytofyx-Citoperm solution for 20 min at 4 °C, in order to permit the cellular permeabilization. After the incubation the cells were washed twice and incubated with 300 μl of PBS 1X-BSA 1% (Bovine Serum Albumin, Sigma-Aldrich, Saint Louis, MO, USA) solution for 1 h at room temperature in darkness. The BSA has the capacity to block the aspecific binding sites. The cells were then collected by centrifugation and incubated with 30 μl of Hemoglobin β-PE (PE-Phycoerythrin) (Santa Cruz Biotechnology, Santa Cruz, CA, USA), diluted 1:10 in PBS 1X – BSA 1%, for about 20 h at 4 °C in darkness. After incubation, the cells were washed with 500 μl of BD Perm/Wash Buffer (BD Biosciences, Franklin Lakes, NJ, USA) diluted 1:10 with water, resuspended with 300 μl of Staining buffer (PBS 1X plus 1% FBS) and transferred to a FACS tube. The analysis was performed with FACScan and the software Cell Quest Pro (Becton-Dickinson, Franklin Lakes, NJ, USA).

### Western blotting

Treated or untreated K562-derived cells were lysed in ice cold RIPA buffer (10 Mm Tris-HCl, pH 8.0, 0.5 mM EDTA, 150 mM NaCl, 1% v/v NP40, 0.1% v/v SDS, 5 mg/ml DeoxyCholic acid, 1 mM DTT, 2 mM PMSF, 2 mM Na_3_VO_4_, 10 mM NaF, 1 μg/ml Leupeptin, 1 μg/ml Aprotinin). Briefly, K562-derived cells (8 × 10^6^ cells) were collected and washed twice with cold PBS (Phosphate-Buffered Saline, Lonza-Biowhittaker, Basel, Switzerland). Cellular pellets were then resuspended with 400 μl of cold RIPA buffer, incubated on ice for 20 min and subjected to 5 cycles of freezing – thawing. Samples were finally centrifuged at 14.000 x g for 3 min at 4 °C and the supernatant cytoplasmic fractions were collected and immediately frozen at − 80 °C. Protein concentration was determined using Pierce™ BCA Protein Assay Kit (Thermo Fisher Scientific, Rockford, USA). 10 μg of cytoplasmic extracts were denatured for 5 min at 98 °C in 1X SDS sample buffer (62,5 mM Tris-HCl pH 6.8, 2% SDS, 50 mM Dithiotreithol (DTT), 0.01% bromophenol blue, 10% glicerol) and loaded on SDS-PAGE gel (10 cm × 8 cm) in Tris-glycine Buffer (25 mM Tris, 192 mM glycine, 0.1% SDS). The Pierce^®^ 3-Color Prestained Protein Molecular Weight Marker (Thermo Fisher Scientific, Waltham, MA, USA) was used as standard to determine molecular weights. The electrotransfer to 20 μm nitrocellulose membrane (Thermo Fisher Scientific, Waltham, MA, USA) was performed over-night at 360 mA and 4 °C in electrotransfer CAPS buffer (10 mM CAPS pH 10.5, 10% methanol). The membrane was prestained in Ponceau S Solution (Sigma Aldrich, St Louis, MO, USA) to verify the transfer, washed with 25 ml Tris-buffered saline (TBS) (10 mM Tris-HCl pH 7.4, 150 mM NaCl) for 10 min at room temperature and incubated in 30 ml of blocking buffer (TBS, 0.1% Tween-20, 5% nonfat dry milk) for 2 h at room temperature. Membranes were washed three times for 5 min each with 30 ml of TBS/T (TBS, 0.1% Tween-20) and incubated with primary rabbit monoclonal antibody against UPF1 (1:15.000) (Origene, Rockville, MD,) in 15 ml primary antibody dilution buffer (TBS, 0.1% Tween-20, 5% BSA) with gentle agitation over-night at 4 °C. The day after, membranes were washed three times for 5 min each with 30 ml of TBS/T and incubated in 15 ml of blocking buffer, in gentle agitation for 2 h at room temperature, with an appropriate HRP-conjugated secondary antibody (1:2.000). Finally, after three washes each with 30 ml of TBS/T for 5 min, membranes were incubated with 10 ml LumiGLO^®^ (0.5 ml 20X LumiGLO^®^, 0.5 ml 20X Peroxide and 9.0 ml Milli-Q water) (Euroclone Pero, Milan, Italy) for 5 min at room temperature and exposed to x-ray film (Thermo Fisher Scientific, Waltham, MA, USA). As necessary, after stripping procedure using the Restore™ Western Blot Stripping Buffer (Thermo Fisher Scientific, Waltham, MA, USA) membranes were reprobed with primary and secondary antibodies. X-ray films for chemiluminescent blots were analyze by Gel Doc 2000 (Bio-Rad Laboratoires, Hercules, CA, USA) using Quantity One software to elaborate the intensity data of our specific protein targets. Protein p70 was used as reference for the normalization.

## Results

### Production of the lentiviral vectors carrying UPF1 or scramble shRNA constructs

**Fig. 1:**
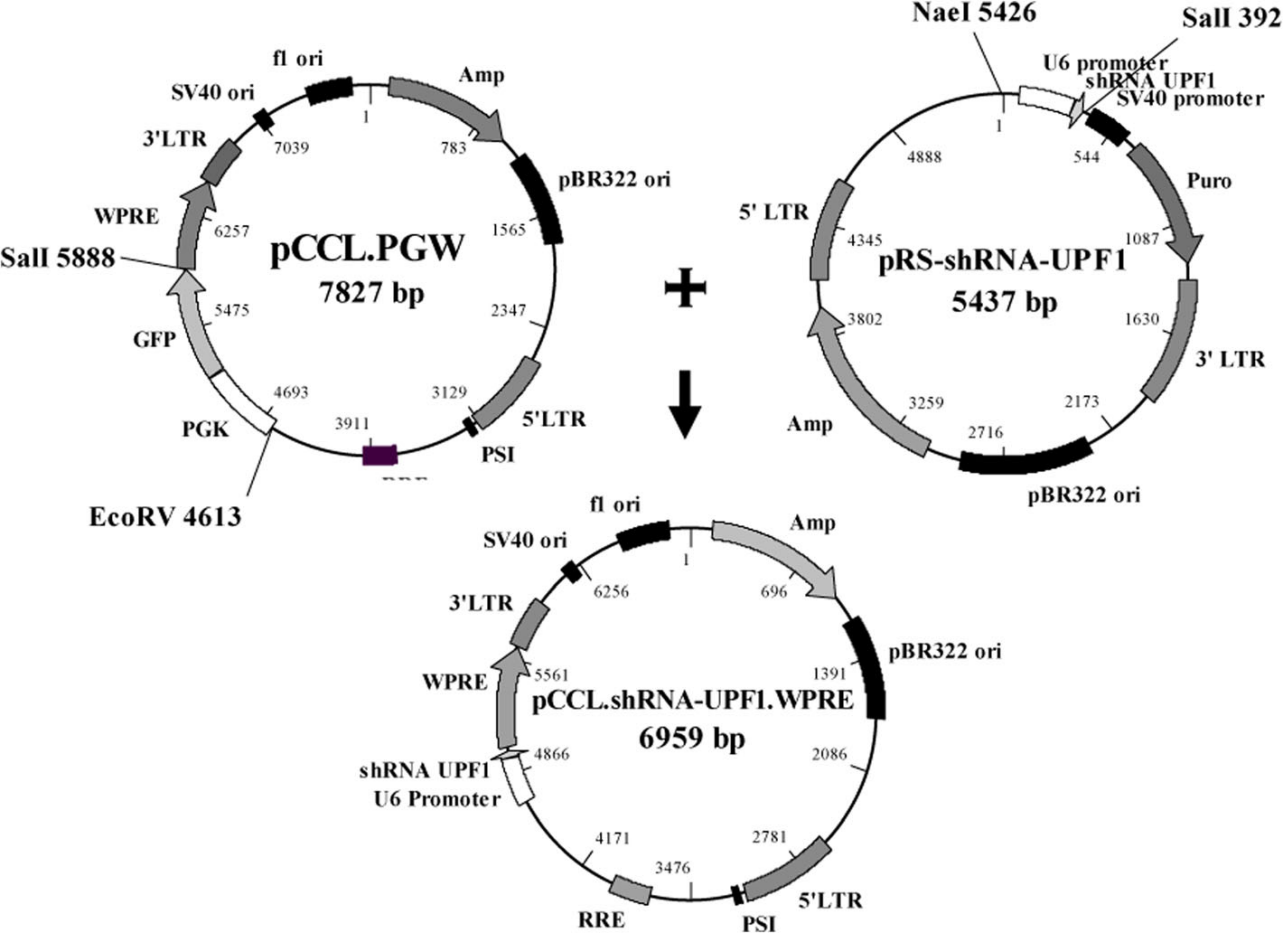
Cloning strategy for the production of the pCCL.shRNA-UPF1. WPRE lentiviral vector. The pRS-shRNA-UPF1 construct was subjected to enzymatic digestion by the restriction enzymes NaeI and SalI with the purpose to isolate the 410 bp insert containing U6 promoter and UPF1 shRNA.pCCL.PGW vector, as the result of cutting by the SalI and EcoRV endonucleases, was deleted of a region containing the GFP gene and the relative PGK promoter. That region was replaced by the 410 bp insert deriving from the pRS-shRNA-UPF1, obtaining the final vector pCCL.shRNA-UPF1.WPRE

To produce a cellular model with a stable suppression of the NMD, a third generation lentiviral vector, carrying a shRNA sequence able to recognize and suppress the UPF1 mRNA, was developed. Figure 1 shows the experimental strategy followed to generate the UPF1 shRNA lentiviral vector. This strategy planned the use of the commercial retroviral vector pRS-shRNA-UPF1 and the lentiviral construct pCCL.PGW [17]. The first one contains an shRNA sequence under the control of the U6 promoter, essential for the shRNA proper expression. We could not directly use this vector due to the presence of the resistance to puromycin, which is counterproductive in the case of treatment with aminoglycoside. The pCCL.PGW construct contains the GFP gene under the control of the constitutive promoter PGK, which had to be eliminated because the target cells already express green fluorescence and we decided not to introduce additional variables among original cells, which were our negative control, and the UPF1 silenced cell clones. So, the fragment SalI-EcoRV obtained by the digestion of the pCCL.PGW vector, containing the GFP gene and its promoter, was replaced by the fragment SalI-NaeI of the pRS-shRNA-UPF1 construct, containing the shRNA sequence and the U6 promoter, obtaining in this way the pCCL.shRNA-UPF1.WPRE lentiviral vector. To confirm the presence and the exact sequence of the shRNA construct, we sequenced the final vector using primers able to amplify the shRNA and U6 promoter region.

The same strategy was followed to produce a lentiviral vector containing a scramble shRNA (named pCCL.shRNA-scramble.WPRE), to be used to produce cell clones used as negative control.

The most efficient UPF1 sequence in suppressing UPF1 expression was previously identified by testing three different siRNAs in our cells (Additional file 1: Figure S1).

### Production and characterization of UPF1 silenced K562.β^0^39 and βwt clones

In order to produce a cellular model of β^0^39 thalassemia with the mechanism of the NMD suppressed, we transduced the pCCL.shRNA-UPF1.WPRE construct into β^0^39.m5 clone, previously generated and derived from K562 cell line [8].

Transduced cells were subsequently cloned by limited dilution so as to obtain clonal populations of cells with the same characteristics. To ensure that the effects on the suppression of UPF1 were specific, the β^0^39.m5 clones were transduced with the pCCL.shRNA-scramble.WPRE construct and after cloned. To obtain read-through activity controls, we transduced both the lentiviral vectors into the βwt.wt3 clone previously generated [8], achieving, after limiting dilution, several cell clones. The βwt.wt3 and β^0^39.m5 clones were previously generated by stable transduction with a lentiviral vector carrying wild-type or mutated β^0^39 globin genes under the control of a minimal LCR region in K562 cells [8]. The clones wt3 and m5 were chosen because they displayed similar levels of accumulation of β globin mRNA, facilitating, therefore, a correct interpretation of the results obtained after treatment with read-through molecules [8].

Once identified the positive cell clones by PCR, amplifying a specific lentiviral sequence, it was necessary to characterize them from the point of view of DNA, RNA and proteins, with the aim to evaluated: (1) the number of vectors which are integrated; (2) the UPF1 gene expression, to verify the effective UPF1 suppression shRNA-mediated; (3) the production of the β^0^39 mRNAs, the presence of which is essential for an ex vivo model of β^0^39 thalassemia and whose accumulation is necessary to evaluate the effects of read-through inducers; (4) the effective reduction of the UPF1 protein content.

**Fig. 2:**
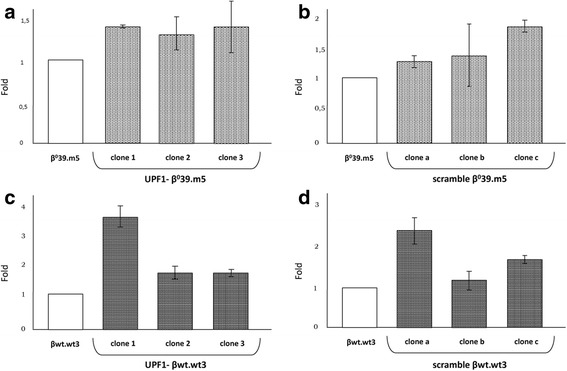
Quantification of the insertion of pCCL.shRNA-UPF1.WPRE and pCCL.shRNA-scramble.WPRE vectors in the obtained clones. Analysis of the integration of pCCL.shRNA-UPF1.WPRE vector in β^0^39.m5 (**a**) and βwt.wt3 (**c**) derived clones and of pCCL.shRNA-scramble.WPRE construct in β^0^39.m5 (**b**) and βwt.wt3 (**d**) clones. Histograms show the integration of the lentiviral vectors understood as fold compared to insertions already present in the respective original cells, used as reference samples. The data represent the averages ± SD of three independent determinations

The analysis of the number of integrations of the lentiviral vectors containing the shRNA constructs in the β^0^39.m5 and βwt.wt3 clones was performed on genomic DNA, isolated from the different clones, by qRT-PCR, amplifying the insertion. The results are shown in Fig. 2 and are reported as a fold of integrated vectors respect to the corresponding control cells (β^0^39.m5 (A-B) and βwt.wt3 (C-D)) set at 1. It was not possible to quantify the exact amount of integrated constructs for two reasons: (1) because the original cells already contain several integrated lentiviral vectors [8]; (2) because the K562 cells do not present a normal diploid karyotype, as they derived from a pleural effusion of a patient with chronic myelogenous leukemia, which is a clonal malignant disorder of the pluripotent hematopoietic stem cells, presenting complex dislocations, with three, four or five chromosomes involved. So, in K562 most chromosomes are in three or more copies [11]. In fact the integrations have taken place in a variable manner in the various clones: from a fold of 1.2 to a fold of 3.6 (Fig. 2).

**Fig. 3:**
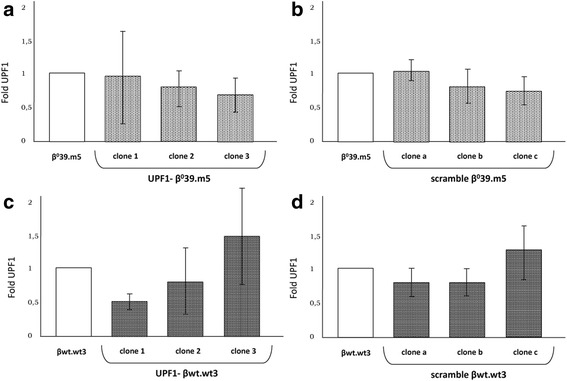
Quantification of the UPF1 mRNA content in UPF1- and scramble clones. Histograms show the UPF1 mRNA content in UPF1- (**a**) and scramble (**b**) β^0^39.m5 clones and UPF1- (**c**) and scramble (**d**) βwt.wt3 clones and in their original cell lines, β^0^39.m5 and βwt.wt3, used as reference samples. The data were obtained by Real Time qRT-PCR using expression assays for UPF1 and GAPDH cDNAs, the last of which was used for the normalization. The data represent the averages ± SD of three independent determinations

Of great importance was to verify whether the integration of the constructs induced the partial UPF1 suppression analyzing UPF1 mRNA levels by qRT-PCR. In Fig. 3 are reported the content of UPF1 transcripts of each clone expressed as a fold respect to the relative control cell line (β^0^39.m5 (A-B) and βwt.wt3 (C-D)) set at 1. Among K562-derived clones transduced with UPF1 shRNA, those with the greater suppression of UPF1 were clones 3 (0.7) and 1 (0.5), respectively, while clones transduced with the scramble shRNA whose expression of UPF1 deviated less from the controls are a (1.1) and c (1.2).

**Fig. 4:**
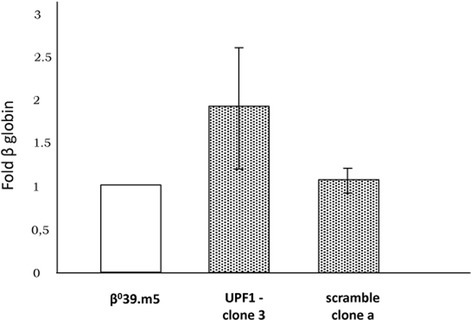
Quantification of the β globin mRNA content in UPF1- and scramble β^0^39.m5 clones. Histograms show the UPF1 mRNA content in UPF1- clone 3, scramble clone a and in the original cell line β^0^39.m5, used as reference samples. The data were obtained by Real Time qRT-PCR using expression assays for β globin and GAPDH cDNAs, the last of which was used for the normalization. The data represent the averages ± SD of three independent determinations

As the suppression of UPF1 can increase the accumulation of nonsense globin transcripts but not of normal ones, we decided to determine the expression of the β globin gene only in clones obtained from the transduction of β^0^39.m5 cells (Fig. 4). By qRT-PCR, we determined that in UPF1- clone 3 the content of β globin mRNA was about twice (1.92) respect to the control cells, while the scramble clone a did not show any significant difference from the control (1.1).

**Fig. 5:**
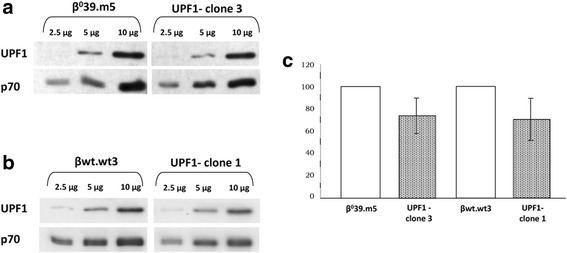
Evaluation of the content of UPF1 protein in UPF1- clones. The UPF1 protein accumulation in UPF1- clones 3 (**a**) and 1 (**b**) compared to the respective control cells, β^0^39.m5 and βwt.wt3, was determined by Western Blotting using scalar quantities of protein extracts and monoclonal antibodies against UPF1 and p70 proteins. The image obtained was analysed by densitometry, which evaluated the relative amount of protein staining and quantified the results in terms of optical density: the histogram shows the reduction in UPF1 protein content in clone 3 and 1, meant as a percentage compared to the corresponding control cells (**c**). Values were normalized using p70 protein. The data represent the averages ± SD of three independent determinations

The results of the qRT-PCR on UPF1 showed a reduction of the transcripts content in K562-derived clones due to suppression mediated by the shRNA against UPF1 mRNA. Because the reduction of the transcripts does not necessarily imply a reduction of the proteins, for the UPF1- clones showing the most reduction in UPF1 expression (Fig. 3a, c), we evaluated the protein content by Western Blotting (Fig. 5). In Fig. 5a-b are reported example blots of the clones 3 and 1 compared to the relative control cell line β^0^39.m5 and βwt.wt3 respectively. The histogram in Fig. 5c shows the UPF1 protein content in both clones as a percentage compared to the corresponding control cells: 74.3 and 71.6% for clone 3 and clone 1, respectively. These data confirm the suppressing action mediated by the shRNA against UPF1 transcripts and a likely subsequent reduction of the NMD.

### Effects of Geneticin (G418) on β globin production in UPF1 silenced K562.β°39 and βwt clones

**Fig. 6:**
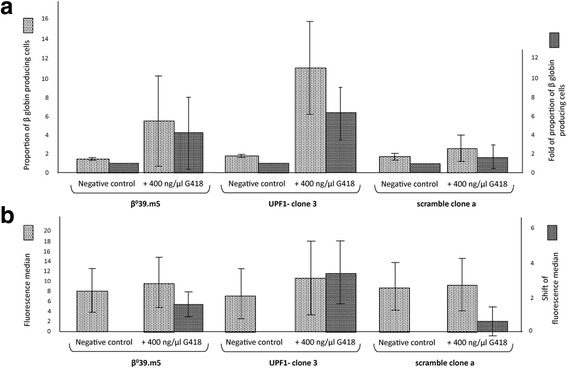
Effects of geneticin (G418) on the β globin production in UPF1- clone 3, scramble clone a and relative original cell line β^0^39.m5. After a 3 day incubation with 400 ng/μl G418, cells were labeled with the monoclonal antibody Hemoglobin β-PE and analysed by FACS. Histogram **a** shows the proportion of β globin producing cells and the fold of that proportion in β^0^39.m5, clone 3 and clone a cells, untreated and treated with 400 ng/μl G418. Histogram **b** shows the fluorescence median of all the samples and its shift after treatment. The data represent the averages ± SD of three independent experiments

**Fig. 7:**
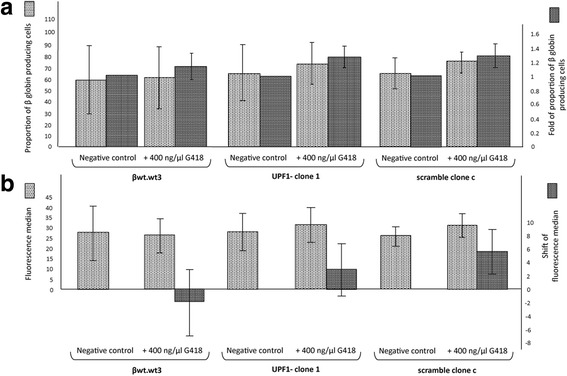
Effects of geneticin (G418) on the β globin production in UPF1- clone 1, scramble clone c and relative original cell line βwt.wt3. After a 3 day incubation with 400 ng/μl G418, cells were labelled with the monoclonal antibody Hemoglobin β-PE and analysed by FACS. Histogram A shows the proportion of β globin producing cells and the fold of that proportion in K562.βwt.wt3, clone 1 and clone c cells, untreated and treated with 400 ng/μl G418. Histogram B shows the fluorescence median of all the samples and its shift after treatment. The data represent the averages ± SD of three independent experiments

Since geneticin (G418) had previously shown good read-through efficiency on β^0^39 globin transcripts in β^0^39.m5 clone derived from K562 cell line [8, 17], we decided to use this aminoglycoside initially to check its read-through activity in the presence of a partial suppression of UPF1. For this reason, we treated the β^0^39.m5 and βwt.wt3 control cell lines and their UPF1- clones 3 and 1 (for shRNA-UPF1) and scramble clones a and c (for shRNA-scramble) with 400 ng/μl geneticin for 72 h, after that we fixed, permeabilized and labeled cells with an antibody against β globin (PE-conjugated Hemoglobin β (37-8*)* antibody) followed by FACS analysis. We used this aminoglycoside concentration because we had previously verified that is the most efficient [8, 17]. The obtained data relative to the β^0^39.m5 and βwt.wt3 clones are reported in Figs. 6 and 7, respectively.

The histograms in Figs. 6a and 7a show the averages (± SD) of data, obtained in three independent experiments, relating to the percentage of fluorescent cells (producing β globin chains) and the relative increase in the treated samples compared to negative controls, while the histograms in Figs. 6b and 7b show the averages (± SD) of the fluorescence median of all the samples and its shift after treatment. In the presence of G418, the β^0^39.m5 control cells show an increase in the percentage of fluorescent cells equal to 5.4%; while the derived UPF1- clone 3 and scramble clone a obtained an increase of 10.9 and 2.4% respectively (Fig. 6a). Similarly the shift of the fluorescence median corresponds to 1.57 for the control cells, 3.36 for clone 3 and 0.55 for clone a (Fig. 6b).

As it regards the βwt clones, the treatment with geneticin did not produce any significant variation in the percentage of fluorescent cells nor even in the shift of the median (Fig. 7). In this way we were able to exclude inductive effects on the expression of β globin due to the G418 treatment, the partial suppression of the NMD and the presence of shRNA.

Whereas the fluorescence corresponds to the presence of β globin chains, we can not only confirm that the geneticin is able to induce read-through of premature stop codon caused by the β^0^39 nonsense mutation, resulting in an increased production of β globin chains, but we can say that this aminoglycoside induces the read-through more efficiently in the UPF1- clone 3, derived from β^0^39.m5 cell line, reflecting the fact that in the presence of a partial suppression of NMD, the increase of the amount of nonsense transcripts enables the production of a greater amount of full β globin chains.

**Fig. 8:**
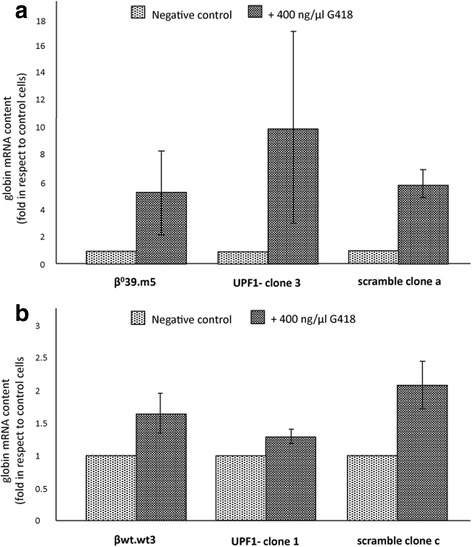
Effects of geneticin (G418) on the content of β globin mRNA in β^0^39 and βwt clones. Histograms show the β globin mRNA content in β^0^39 (**a**) and βwt (**b**) clones, UPF1 or scramble silenced, and in the original cell lines, β^0^39.m5 and βwt.wt3 respectively, untreated and treated with 400 ng/μl G418. The data were obtained by Real Time qRT-PCR using expression assays for β globin and GAPDH cDNAs, the last of which was used for the normalization. The data represent the averages ± SD of three independent experiments

Once proved the major effects of G418 as translational corrector of the β^0^39 nonsense mutation in the presence of a partial suppression of NMD, we wondered if the treatment with the aminoglycoside in this condition prevents more efficiently the decrease in β^0^39 globin mRNA abundance attributable to NMD. In a previous work we have verified that geneticin, binding the decoding centre of the ribosome and decreasing the accuracy requirements for codon-anticodon pairing, suppresses stop codon, preventing the recognition of the nonsense transcript by the NMD [17]. In this respect, we performed a qRT-PCR on the RNA isolated from β^0^39.m5 and βwt.wt3 clones (UPF1 or scramble silenced) untreated and treated with 400 ng/μl G418. The obtained data are presented in Fig. 8 as averages ± SD of three independent experiments. After the treatment with geneticin, the β^0^39.m5 control cell line and its scramble clone a present a fold in β globin transcript content equal to 5.28 and 4.71, respectively, while the UPF1- clone 3 shows a fold of 10.01 (Fig. 8a), suggesting that the partial suppression of the NMD allows a greater rescue of the mutated transcripts mediated by G418. The treatment of the βwt.wt3 clones does not induce a significant increase in β globin content as shown in Fig. 8b, confirming that geneticin has no major effects on the transcription, processing and stability of the wild-type β globin mRNA.

The characterized UPF1- clone 3 derived from β^0^39 globin K562 cell line (β^0^39.m5) has been tested also for other aminoglycosides (including tobramycin and gentamicin), confirming its possible application as cellular model system for the screening of read-through molecules (Additional file 1: FigureS2).

## Discussion

While read-through activity introduced a great hope in the case of several pathologies due to stop codon mutations, this approach is so far not very efficient, due to several unresolved problems, among which the NMD decay of mRNAs carrying stop-codon mutations is the most relevant. The NMD pathway degrades mRNAs containing PTCs, thereby limiting the expression of potentially deleterious truncated proteins. This positive action turns out to be a very important limit for read-through correction in several diseases, such as β thalassemia and cystic fibrosis [18, 19]. This is the reason for recent studies focusing on the identification of low-molecular weight molecules potentiating the activity of read-through molecules, thanks to their NMD inhibition activity [10, 20–22]. Read-through molecules can increase the full-length protein synthesis, while NMD inhibition are expected to increase the levels of mRNA even if they carry a stop codon mutation. Strategies promoting both read-through activity and RNA stabilization (either using molecules exhibit these two activities, or using a read-through corrector and a NMD inhibitor in combination) are expected to be more efficient to promote high abundance of mRNA available for translation and correct the effect of stop codon mutations.

While several molecules designed to enhance translational read-through have been reported to concomitantly inhibit NMD efficiency, experimental tools for systematic identification of molecules preferentially exhibiting translational readt-hrough activity are lacking. This is a key issue and screening systems allowing the easy identification of read-through molecules expected to be potentiated by NMD inhibitors are of great interest. For instance, a system for coordinated analysis of translational read-through and nonsense-mediated mRNA decay has been recently proposed by Baker and Hogg [23] based on three different RNA elements (MLVPK, CTFVHP and a nonsense mutation associated with junctional epidermolysis bullosa) [23].

Here we have produced and characterized the UPF1- clone 3 derived from β^0^39 globin K562 cell line (β^0^39.m5), where UPF1, a essential phosphoprotein for NMD function, has been partially suppressed. This cellular model presented a stable modulation of the NMD by the transduction of a lentivirus vector carrying shRNA sequence able to recognize and suppress the UPF1 mRNA. Our system showed a partial suppression of the NMD, allowing a greater rescue of the mutated transcripts mediated by molecules promoting read-through activity as aminoglycosides (such as G418, gentamicin and tobramycin).

## Conclusions

The combination of read-through activity and NMD inhibition should be considered for high efficiency in the correction of PTCs. In facts the NMD attenuation could increase the abundance of PTC-containing mRNAs providing higher levels of functional protein produced by PTC suppression and a greater therapeutic benefit.

We propose this system for the screening of molecules exhibiting preferential read-through activity to be used in combination with NMD inhibitors.

## Additional file

Additional file 1: (**Figure S1**). To be sure to clone an shRNA really efficient in suppressing UPF1, we first tested three different siRNAs, in a transient way using siPORT™ NeoFX™ Transfection Agent (Thermo Fisher Scientific, Waltham, MA, USA). After 2 days incubation, we evaluated the expression of UPF1 by Real Time qPCR assay. The results show the UPF1 fold expression in presence of each siRNA compared to control cells transfected with a scramble siRNA. The UPF1c showed the best suppression effect (**Figure S1**). (**Figure S2**). The UPF1- clone 3 derived from β^0^39 globin K562 cells (β^0^39.m5) has been tested with different aminoglycosides (geneticin, tobramycin and gentamicin), confirming its application as cellular model for screening of read-through molecules. As far as tobramycin, it was able to induce read-through activity with a complete NMD suppression in yeast models [24, 25]. On the other hand, the read-through activity of gentamicin is firmly established [6, 19]. For these reasons, we treated the cells with 400 ng/μl geneticin (G418), 1400 ng/μl gentamicin or 1000 ng/μl tobramycin for 72 h [6, 24]. Then the cells were labeled with an antibody against β globin, followed by FACS analysis (see Methods section). **Figure S2** shows the averages (± SD) of the data obtained in three independent experiments, representing the percentage of fluorescent cells (producing β globin chains) and the relative increase in the treated samples compared to negative controls. With G418, the β^0^39.m5 control cells show a 5.4% increase in the proportion of fluorescent, which was 10.9% in the derived UPF1- clone 3 (**Figure S2A** and Fig. 6a). After gentamicin and tobramycin treatment, the β^0^39.m5 control cells displayed an increase of 3.3 and 1.2% in the proportion of fluorescent cells, respectively. While in the UPF1- clone 3, the increase was 8.9 and 4.7% after gentamycin or tobramycin treatment (**Figures S2B-C**). (DOCX 635 kb)

## Abbreviations

NMD: nonsense-mediated mRNA decay
PTC: premature termination codon
