# Supplementary material for: UPF1 silenced cellular model systems for screening of read-through agents active on β039 thalassemia point mutation
Source: BMC Biotechnol. 2018 May 15;18:28. doi: 10.1186/s12896-018-0435-0 (PMC5952824; doi:10.1186/s12896-018-0435-0)
Supplement: Supplementary file 1 — (Figure S1). To be sure to clone an shRNA really efficient in suppressing UPF1, we first tested three different siRNAs, in a transient way using siPORT™ NeoFX™ Transfection Agent (Thermo Fisher Scientific, Waltham, MA, USA). After 2 days incubation, we evaluated the expression of UPF1 by Real Time qPCR assay. The results show the UPF1 fold expression in presence of each siRNA compared to control cells transfected with a scramble siRNA. The UPF1c showed the best suppression effect (Figure S1). (Figure S2). The UPF1- clone 3 derived from β039 globin K562 cells (β039.m5) has been tested with different aminoglycosides (geneticin, tobramycin and gentamicin), confirming its application as cellular model for screening of read-through molecules. As far as tobramycin, it was able to induce read-through activity with a complete NMD suppression in yeast models [24, 25]. On the other hand, the read-through activity of gentamicin is firmly established [6, 19]. For these reasons, we treated the cells with 400 ng/μl geneticin (G418), 1400 ng/μl gentamicin or 1000 ng/μl tobramycin for 72 h [6, 24]. Then the cells were labeled with an antibody against β globin, followed by FACS analysis (see Methods section). Figure S2 shows the averages (± SD) of the data obtained in three independent experiments, representing the percentage of fluorescent cells (producing β globin chains) and the relative increase in the treated samples compared to negative controls. With G418, the β039.m5 control cells show a 5.4% increase in the proportion of fluorescent, which was 10.9% in the derived UPF1- clone 3 (Figure S2A and Fig. 6a). After gentamicin and tobramycin treatment, the β039.m5 control cells displayed an increase of 3.3 and 1.2% in the proportion of fluorescent cells, respectively. While in the UPF1- clone 3, the increase was 8.9 and 4.7% after gentamycin or tobramycin treatment (Figures S2B-C). (DOCX 635 kb) [file 12896_2018_435_MOESM1_ESM.docx]

**Additional file 1**

*Identification of the siRNA with the greater efficacy in suppressing UPF1 mRNA*

To be sure to clone an shRNA really efficient in suppressing UPF1 expression, we previously tested in our cells three different siRNAs, in a transient way (**Fig. 1s**). These siRNAs bind UPF1 mRNA in different regions as shown in **Fig. 1sA**. We transfected the siRNAs in our target cells using siPORT™ NeoFX™ Transfection Agent (Invitrogen – Life Technologies) with a final concentration of 800 nM. After 2 days incubation we evaluated the expression of UPF1 by Real Time qPCR assay. In **Fig. 1sB** are reported the results of a preliminary assessment: the histogram shows the folds of the UPF1 expression in presence of each siRNA compared to control cells transfected with a scramble siRNA. The UPF1c showed the best suppression effect and so we decided to focalize our attention on it and we further evaluated its effects on target cells (**Fig. 1sC**), confirming its action in UPF1 suppression.


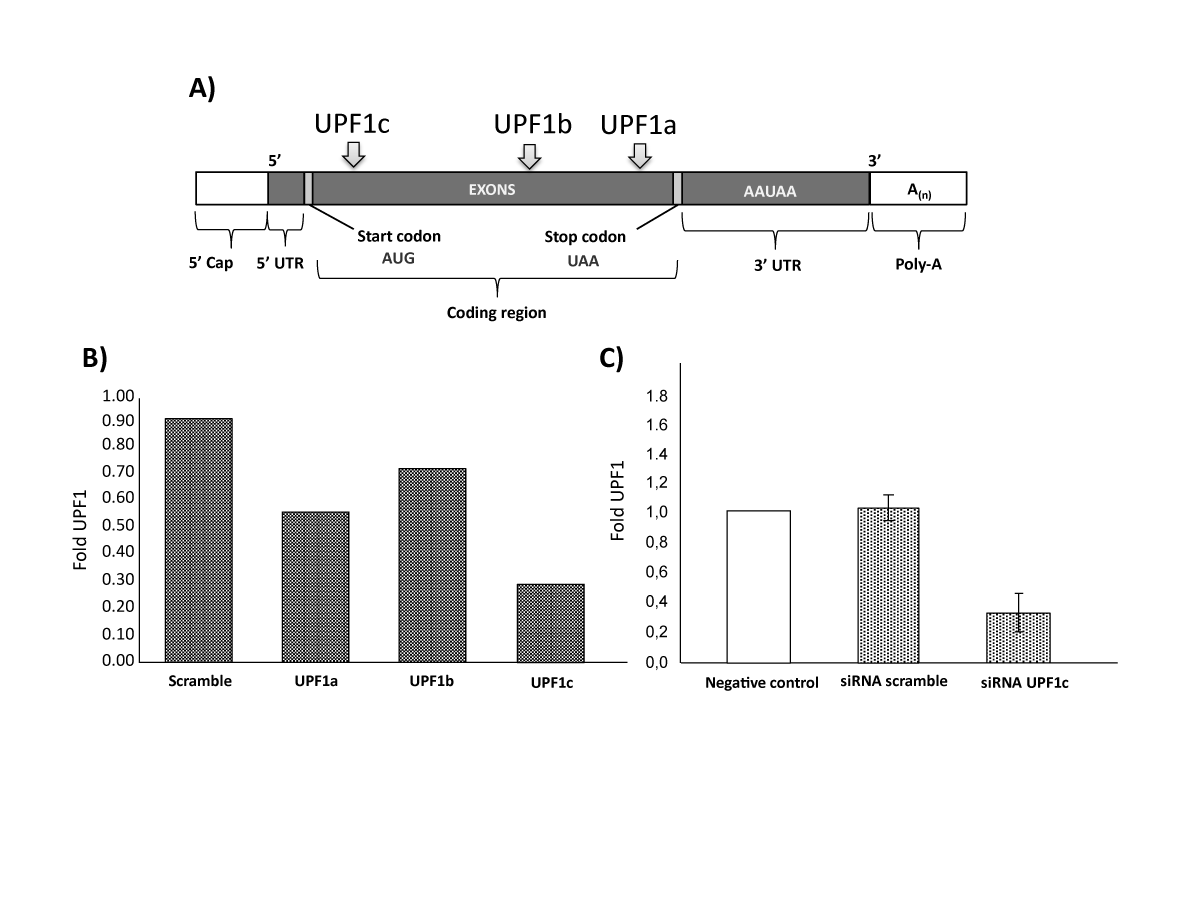


**Fig. 1S** Identification of the siRNA with the greater efficacy in suppressing UPF1 mRNA. A) Schematic rappresentation of the UPF1 gene; arrows indicate the binding of the three siRNAs. B) Histogram showing the UPF1 mRNA content of the K562.β^0^39.m5 cells after the transfection of scramble, UPF1a, UPF1b and UPF1c siRNA. UPF1 siRNA transfection into K562-derived cells (K562.β^0^39.m5 and K562.βwt.wt3) were carried out using siPORT™ NeoFX™ Transfection Agent (Invitrogen, Life Technologies). Cationic liposomes and siRNAs 800 nM were added to the cells and after 24 hours at 37°C, the transfection was repeated. Finally the cDNA was extracted using Cells-to-cDNA™ II kit (Ambion, Life technologies) after 24 hours. C) Histogram showing the UPF1 mRNA content of the K562.β^0^39.m5 cells transfected with Scramble and UPF1c siRNA as averages ± SD of three independent experiments.

*Effects of aminoglycosides on β-globin production in UPF1 silenced K562.β^0^39 clones*

The characterized UPF1- clone 3 derived from β^0^39 globin K562 cell line (β^0^39.m5) has been tested with different aminoglycosides (geneticin, tobramycin and gentamicin), confirming its application as cellular model for screening of read-through molecules (Fig. 2s).

While according with the available reports present in the literature tobramycin exhibits a low read-through activity [25], Altamura et al. [26] recently demonstrated that this aminoglycoside was able to induce in yeast models read-through activity with a total NMD suppression. On the other hand, the read-through activity of gentamicin is widely reported in literature [6,19], significantly increased in the NMD absence. For these reasons, we treated the UPF1- clone 3 and its β^0^39.m5 control cell line with 400 ng/µl geneticin (G418), 1400 ng/µl gentamicin or 1000 ng/µl tobramycin for 72 hours. Then the cells were fixed, permeabilized and labeled with an antibody against β-globin (PE-conjugated Hemoglobin β (37-8*)* antibody), followed by FACS analysis. These chosen aminoglycoside concentrations were based on the literature [6,25]. The histograms in Fig. 2s show the averages (± SD) of the data obtained in three independent experiments, representing the percentage of fluorescent cells (producing β-globin chains) and the relative increase in the treated samples compared to negative controls. In the presence of G418, the β^0^39.m5 control cells show a 5.4% increase in the proportion of fluorescent, which was 10.9% when the derived UPF1- clone 3 was empolyed (Fig. 2sA and Fig. 6A). After gentamicin and tobramycin treatment, the β^0^39.m5 control cells displayed an increase of 3.3% and 1.2% in the proportion of fluorescent cells, respectively. When the derived UPF1- clone 3 was employed the increase after gentamycin and tobramycin treatment was 8.9% and 4.7%, respectively (Fig. 2sB-C).


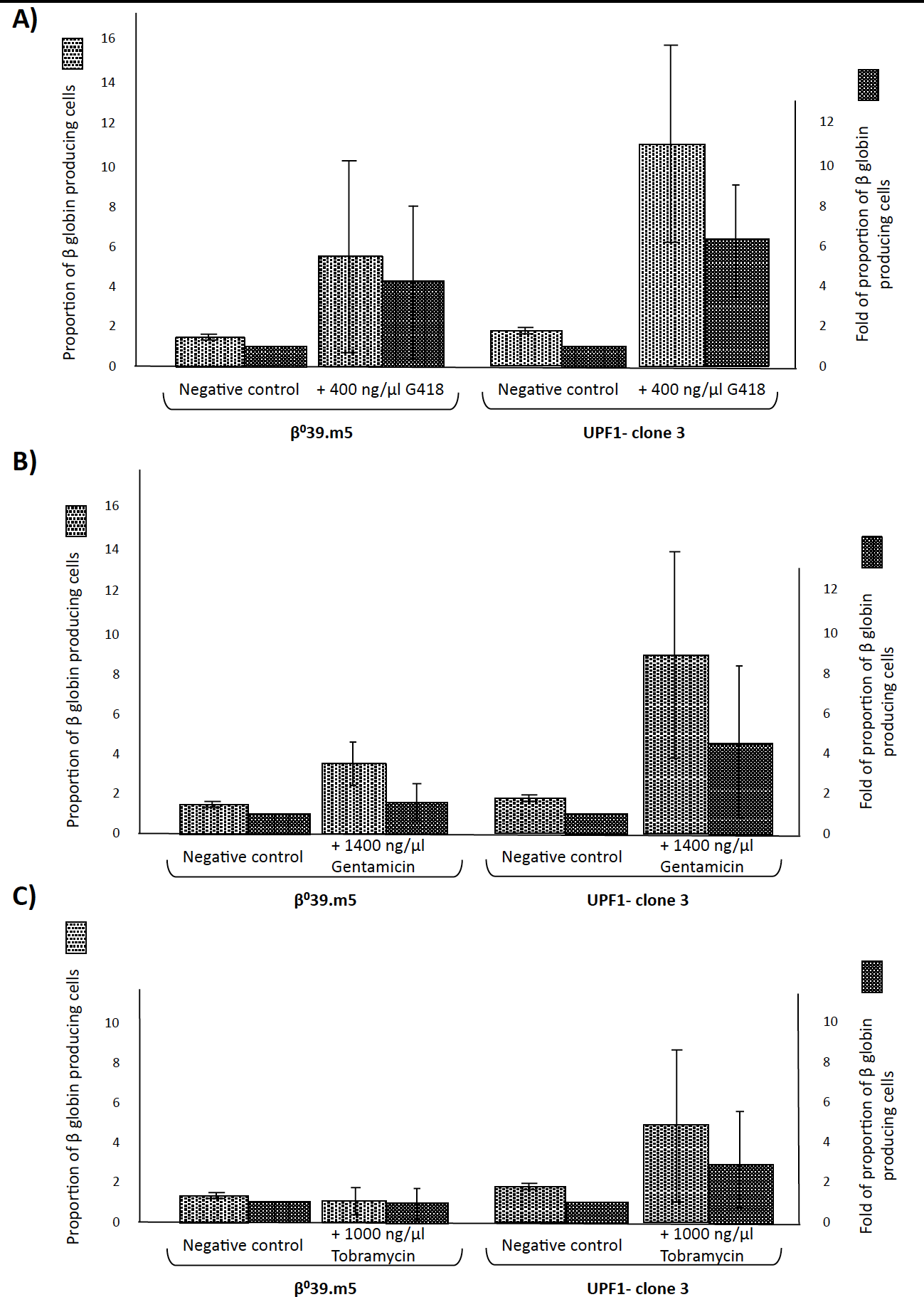


**Fig. 2S.** Effects of geneticin (G418), gentamicin, tobramycin on the β globin production in UPF1- clone 3 and relative original cell line β^0^39.m5. After a 3 day incubation with 400 ng/μl G418, 1400 ng/μl gentamicin or 1000 ng/μl tobramycin cells were labeled with the monoclonal antibody Hemoglobin β-PE and analysed by FACS. Histograms show the proportion of β globin producing cells and the fold of that proportion in UPF1- clone 3 and β^0^39.m5 control cell line untreated and treated with G418 (A), gentamicin (B) or tobramycin (C). The data represent the averages ± SD of three independent experiments.
